# Can *O*-GIcNAc Transferase (OGT) Complex Be Used as a Target for the Treatment of Hematological Malignancies?

**DOI:** 10.3390/ph17060664

**Published:** 2024-05-22

**Authors:** Shiwei Zhuang, Zhimei Liu, Jinyao Wu, Yudan Yao, Zongyang Li, Yanxiang Shen, Bin Yu, Donglu Wu

**Affiliations:** College of Traditional Chinese Medicine, Changchun University of Traditional Chinese Medicine, Changchun 130117, China; 13604414589@163.com (S.Z.); 15843048017@163.com (Z.L.); cczyy6680wjy@163.com (J.W.); 15143163126@163.com (Y.Y.); ccucmlzy1994@163.com (Z.L.); 18347178568@163.com (Y.S.); yubin_2020@163.com (B.Y.)

**Keywords:** OGT, OGT complex, *O*-GlcNAc, hematologic malignancies, epigenetic modification

## Abstract

The circulatory system is a closed conduit system throughout the body and consists of two parts as follows: the cardiovascular system and the lymphatic system. Hematological malignancies usually grow and multiply in the circulatory system, directly or indirectly affecting its function. These malignancies include multiple myeloma, leukemia, and lymphoma. *O*-linked β-N-acetylglucosamine (*O*-GlcNAc) transferase (OGT) regulates the function and stability of substrate proteins through *O*-GlcNAc modification. Abnormally expressed OGT is strongly associated with tumorigenesis, including hematological malignancies, colorectal cancer, liver cancer, breast cancer, and prostate cancer. In cells, OGT can assemble with a variety of proteins to form complexes to exercise related biological functions, such as OGT/HCF-1, OGT/TET, NSL, and then regulate glucose metabolism, gene transcription, cell proliferation, and other biological processes, thus affecting the development of hematological malignancies. This review summarizes the complexes involved in the assembly of OGT in cells and the role of related OGT complexes in hematological malignancies. Unraveling the complex network regulated by the OGT complex will facilitate a better understanding of hematologic malignancy development and progression.

## 1. Introduction

Hematological malignancies, a type of immunocytoma, arise from malignant tumors of the blood and hematopoietic system. These tumors result from the uncontrolled or mutated proliferation and spread of immune cells or cell populations, including myelodysplastic syndrome (MDS), acute myeloid leukemia (AML), chronic myeloid leukemia (CML), and lymphoma [1]. Recent cancer reports show that leukemia and malignant lymphoma rank ninth and tenth in the national mortality rate from malignant tumors [2]. Accumulated research with a basic and clinical focus on leukemia, lymphoma, and multiple myeloma has made tremendous progress in bringing revolutionary breakthroughs in the treatment of hematological tumors, yet many patients have still faced treatment failures due to relapse and drug resistance in the past decade [3]. Hematological malignancies are highly heterogeneous [4], but the emergence of novel chemotherapeutic agents and advances in hematopoietic stem cell transplantation have highly improved the remission and disease-free survival rates of AML patients. However, the high recurrence rate and recurrence refractory of AML is still one of the current research difficulties that experts in this field are concerned about [5]. As a result, hematological malignancies are a serious threat to human life and health. In particular, these malignant tumor cells usually grow and multiply in the circulatory system, directly or indirectly affecting the function of the circulatory system [6]. Leukemia and malignant lymphoma, for example, can lead to generalized lymph node enlargement and splenomegaly [7,8] and cause bone marrow hyperplasia and bone marrow suppression, which in turn intervene in the production of red blood cells, white blood cells, and platelets and affect the normal function of the circulatory system [9].

**Figure 1:**
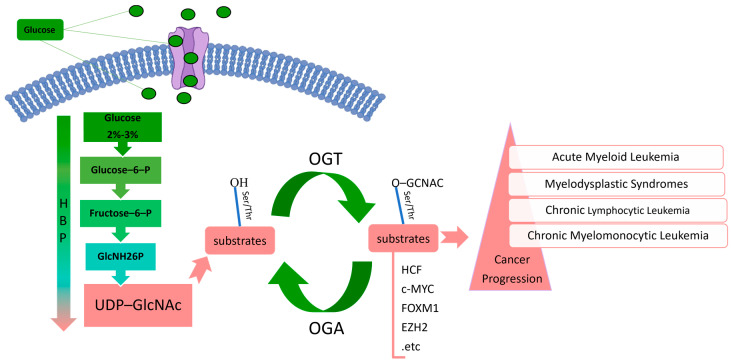
*O*-GlcNAcylation (schematic). About 3% of glucose enters the HBP, providing a UDP-GlcNAc donor for *O*-GlcNAcylation. Under the action of OGT, the GlcNAc monosaccharide moiety of UDP-GlcNAc is added to the Ser/Thr residues of the target protein, resulting in *O*-GlcNAcylation. OGA hydrolyzes *O*-GlcNAc in the target protein. *O*-GlcNAcylation is a nutritional regulator that plays a regulatory role in cancer development. Substrates: HCF [35], c-Myc [20], FOXM1 [36], and EZH2 [37].

*O*-linked β-D-N-acetylglucosamine (*O*-GlcNAc) modification is a post-translational modification (PTM) targeting a variety of proteins located in the nucleus, plasma, as well as mitochondrion named *O*-GlcNAcylation [10,11,12,13]. Highly dynamic reversible changes in *O*-GlcNAcylation are tightly regulated by the sole pair of *O*-GlcNAc transferase (OGT) and *O*-GlcNAcase (OGA) antagonistic enzymes [14,15]. OGT catalyzes the transfer of N-acetyl glucosamine (GlcNAc) from the donor substrate uridine diphosphate N-acetyl glucosamine (UDP-GlcNAc) to the hydroxyl group of serine (Ser) or threonine (Thr) substrate protein residues [16]. OGA reverses this hydrolytic pathway [17]. In recent years, abnormal OGT and *O*-GlcNAcylation have often been observed for a variety of malignancies [18]. Moreover, there is increasing evidence that upregulated *O*-GlcNAcylation has a positive role in immune escape in a variety of cancer cells and regulates the proliferation of tumor cells including colorectal cancer, liver cancer, lung cancer, breast cancer, and even hematological malignancies [19] (as shown in Table 1). For instance, OGT overexpression was observed for pre-B acute lymphoblastic leukemia (pre-B-ALL), and the authors noted that increased OGT-mediated *O*-GlcNAcylation could exacerbate pre-B-ALL through the phosphatidylinositol 3-kinase (PI3K)/protein kinase B (AKT)/c-Myc cascade [20]. On the other hand, approximately 2–3% of the body’s glucose enters the hexosamine biosynthesis pathway (HBP) and is metabolized in combination with amino acids, nucleotides, and fatty acids to produce the donor substrate UDP-GlcNAc for *O*-GlcNAcylation [21]. In order to meet their survival and proliferation needs, tumor cells use glycolysis as the main metabolic pathway to obtain ATP and try to compensate energy metabolism through the HBP pathway [22]. In tumor cells, increased glucose flux through the HBP leads to increased UDP-GlcNAc levels, which in turn prompt an overall increase in *O*-GlcNAcylation [23]. Therefore, *O*-GlcNAcylation is often referred to as a “nutrient sensor” (as shown in Figure 1) that operates in response to changing nutritional and metabolic needs [24]. Asthana et al. showed that targeting glutamine-fructose-6-phosphate transaminase (GFAT) to inhibit the flux of the HBP could induce the differentiation and apoptosis of AML cells (OCI-AML3 and HL-60) [25], thereby indicating the critical role of *O*-GlcNAcylation in cancer proliferation.

**Table 1:** OGT in hematological neoplasms.

Cancer Types	Mechanism	Physiological Effects	Expression	Reference(s)
CLL	OGT-induced c-myc, p53, and Akt *O*-GlcNAcylation.		Decline	[26]
	OGT expression increased.	Increase in resistance.		[27]
pre-B-ALL	OGT-mediated increase in *O*-GlcNAcylation, which in turn activates the PI3K/AKT/c-Myc cascade.	Inhibition of apoptosis.	Rise	[20]
DLBCL	Increased OGT mRNA expression.	Reduction in patients survival.	Decline	[28]
	Silencing OGT results in decreased activity of NF-kB and NFATc.	Growth inhibition.		[28]
CML	OGT-induced STAT5 *O*-GlcNAcylation.	Promotion of cell proliferation.	Decline	[29]
APL	OGT-mediated *O*-GlcNAc-dependent upregulation of LGALS12.	Helping to maintain cell stemness.	Rise	[30]
AML	Inhibition of OGT and OGA, causing *O*-GlcNAcylation imbalance.	Causing inhibition of stem/progenitor cell self-renewal in CD34+ HSPC and AML cells (KG-1) and driving myeloid differentiation.		[31]
	OSMI-1, BADGP, and siRNA-mediated OGT inhibition.	Inducing cell death.	Decline	[32]
	Decreased protein levels of pNF-κB and pAKT and caspase-9/caspase-3 cascade.	Improvement inchemotherapy sensitivity.		[33]
	Increased OGT expression.	Enhancement of resistance to BTZ.		[34]

## 2. OGT

OGT acts as the only glycosyltransferase to catalyze *O*-GlcNAcylation in mammalian cells, and its sequence is highly conserved from Caenorhabditis elegans (*C. elegans*) to humans [38,39]. OGT is encoded by a single gene located on the X chromosome [40,41,42]. Its full length comprises two distinct regions as follows: a superhelical N-terminal structural domain consisting of a tetrapeptide repeat (TPR, 34 amino acids) and a C-terminal catalytic structural domain with glycosyltransferase activity [43]. Among these, the TPR domain is generally considered to be a scaffold that interacts with other proteins and plays a role in determining substrate selection [44], while the catalytic structural domain at the C-terminus mainly binds to the donor UDP-GlcNAc and is responsible for catalytic substrate protein *O*-GlcNAcylation. OGT is an essential enzyme in cellular metabolic pathways [45] that is capable of modifying hundreds of proteins, including cell cycle proteins, those involved in the regulation of biological rhythms, and epigenetically modified genes. On the other hand, OGT also exhibits specialized protease activity and is essential in certain proteolytic processes, such as host cell factor 1 (HCF-1) [46]. In accordance with *O*-GlcNAcylation, OGT was clarified as being upregulated in numerous cancer tissues, including hematologic malignancies. Chronic lymphocytic leukemia (CLL) was the first hematologic malignancy to be noted to exhibit OGT abnormalities [47]. Compared to normal B cells, CLL cells showed a significant increase in OGT activity, which was accompanied by higher *O*-GlcNAcylation of c-myc, p53, and AKT [48]. Relevant studies have shown that high levels of the *O*-GlcNAcylated signal transducer and activator of transcription (STAT) 5 can be observed in human leukemia cell lines, particularly CML cells (Ku812, K562), which promote hematological hematologic malignancy [49]. It is reported that compared to normal B cells, the upregulated OGT level was detected in both diffuse large B-cell lymphoma (DLBCL) cells and tumor tissues. Notably, silencing OGT could suppress the activity of nuclear transcription factor-kappa B (NF-κB) and nuclear factor of activated T cells c1 (NFATc1) as well as cancer cell growth; moreover, consistent with this, increased OGT expression was negatively correlated with patient survival [50]. Consistent with this, both the OGT inhibitor and siRNA-mediated OGT inhibition induce AML cell death [51]. This review summarizes the biological functions of the complexes involved in the assembly of OGT (as shown in Table 2) and their role in hematological malignancies.

## 3. OGT/HCF-1 Complex

HCF-1, which was first isolated from the human herpes simplex virus, is a transcriptional cofactor that regulates the indicated target gene transcription, such as the cell cycle regulatory proteins E2 promoter binding factors (E2Fs) [66], the maintenance of the embryonic stem cell pluripotency factor Ronin [67], and the regulatory proteins for glucose metabolism proliferator-activated receptor-gamma coactivator-1 (PGC-1) [68] and forkhead box O (FOXO) [69], which in turn are involved in the regulation of embryonic stem cell pluripotency, cell cycle progression, cell proliferation, and metabolic stress. Studies have shown that high expression of HCF-1 is a marker for poor prognosis in the treatment of malignancies such as primary prostate tumors, lymphomas, AML, and sarcoid lymphomas [70]. For example, Glinsky et al. showed by Kaplan–Meier analysis that HCF-1 can lead to treatment failure and short survival in patients with malignancies such as DLBCL, sarcoid lymphoma, and AML [71]. The activation of HCF-1 was driven by OGT-mediated proteolysis, of which the threonine domain interacts with the OGT TPR domain, while the cleavage domain interacts with the OGT catalytic domain, and the cleavaged HCF-1_N_ and HCF-1_C_ bind through noncovalent bonds [72,73,74,75]. Furthermore, the cleavaged HCF-1_N_ interacted with OGT and assembled the OGT/HCF-1 complex [76,77]. The HCF-1/OGT complex inhibits the PR-Set7-dependent switch of H4K20 from being monomethyl to dimethylated during mitosis in HELA cells, thereby promoting M-phase progression and the proliferation of cervical cancer cells [52]. Notably, H4K20 dimethylation inhibits p21 expression, thereby accelerating the cell cycle G1/S transition and promoting the growth of CLL cells (K562) [78]. Moreover, the OGT/HCF-1 complex reduces the cytotoxicity of carfilzomib, a clinically used proteasome inhibitor, to myeloma (MM) and T-cell-derived acute lymphoblastic leukemia (T-ALL) cells by increasing the level of nuclear respiratory factor 1 (NRF1) protein [79,80]. In addition, the OGT/HCF-1 complex can synergize with other proteins to form temporary complexes to perform related functions [81]. For instance, HCF-1 could interact with MYC and regulate ribosome biogenesis and mitochondrial function, further promoting the proliferation of lymphoma cells (Ramos) [53]. Based on both MYC and HCF-1 being able to be glycosylated by OGT, it could be supposed that the OGT/HCF-1 complex could synergize with MYC. OSMI-2 (OGT inhibitor) and OGT siRNA-mediated OGT suppression destroyed the interaction of MYC and HCF-1, further decreasing the expression of Cyclin B1 and polo-like kinase 1 (PLK1) [54]. In activated B-cell (ABC) and germinal center B-cell (GCB)—DLBCL cells (SU-DHL-6 and Jeko-1) as well as mantle cell lymphoma (MCL), downregulated cyclin B1 and Polo-like kinase 1 (PLK1) reduced the G2/M phase of cells (Jeko-1), thereby inhibiting cell proliferation [82]. Another HCF/OGT-interacting protein mixed-lineage leukemia (MLL) 5 gene, also known as lysine (K) N-methyltransferase 2 (KMT2E), is essential for the methylation of H3K4 [83]. Downregulated MLL5 decreased the H3K4me3 of the promoter of E2F1and cell cycle-regulated element (CCRE) [84,85]. MLL5 can also be assembled in the OGT/HCF-1/MLL5 complex [55], and OGT-mediated *O*-GlcNAcylation could block ubiquitination and maintain the stability of MLL5 [86]. Meanwhile, the MLL5 protein recruited to the promoter region of the E2F1 gene through binding to HCF-1, which in turn increased histone H3K4me3 levels in the E2F1 promoter and activated transcription of the E2F1 target genes (CyclinA, CDC2, and CDC6) to promote the G1-to-S-phase transition of the HEK293T and HeLa cell cycles [84]. The gene encoding MLL5 is located in the chromosome 7q22 region [87]. It is reported that in AML, acute lymphoblastic leukemia, and MDS, 7q22 fragment (encoding MLL5) deletion was often detected [88,89]. Consistent with this, overall survival (OS) and relapse-free survival (RFS) were longer in patients with high MLL5 expression compared to those with low MLL5 expression [90]. Based on the stabilizing effect of OGT on MLL5 protein levels in the OGT/HCF-1/MLL5 complex and the recruitment of MLL5 by HCF-1, we can speculate on the important role of the OGT/HCF-1/MLL5 complex in hematological malignancies. In summary, the OGT/HCF-1 complex is involved in the regulation of epigenetic modifications and, on this basis, acts synergistically with the related proteins involved in the regulation of the cell cycle, ribosome biosynthesis, mitochondrial function, and gene expression [91].

## 4. NSL Complex

Accompanying other subunits, males absent on the first (MOF) protein, NSL1, NSL2, NSL3, microspherule protein 2 (MCRS2), HCF-1, tryptophan-aspartate repeat domain 5 (WDR5), OGT, and HCF-1 also assembled into the NSL complex [92,93]. The complex subunit population of the NSL complex gives itself a relatively broad substrate specificity, including the lysine (K) 16, K5, and K8 sites of acetylated histone H4 [94]. Further mediated indicated target gene transcription, such as yin-yang 1 (YY1), X-box binding protein 1 (XBP1), and kruppel-like factor 6 (KLF6), affect the fundamental processes in tumor cells, such as cell proliferation, cell cycle, cell autophagy, and apoptosis [95,96]. MLL-AF9 gene defects are formed by reciprocal translocation of t (9;11). Expression of MLL-AF9 fusion genes has been associated with extramedullary tumor infiltration, high recurrence rates, and low survival [97]. An example is P2X7 (P2X7 belongs to the P2X family of purinergic receptors and is aberrantly expressed in various types of malignant tumors, including leukemia) [98]. MOF is a histone 4 lysine 16 (H4K16) acetyltransferase and is a member of the lysine acetyltransferase MYST family, which is named after its founding members, MOZ, YBF2, SAS2, and TIP60, with MOF being a member of the MYST family [31]. MOF is one of the most representative proteins in the MYST family and acts as a cell type-dependent regulator of chromatin status, controlling various cellular processes, such as DNA damage response, cell cycle progression, and the self-renewal and pluripotency of embryonic stem cells, and an RNAi screen has shown that MOF plays a crucial role in MLL-AF9 leukemogenesis [99]. Catalytic subunit MOF deletion leads to overall H4K16ac depletion in the genome of MLL-AF9 (MA9) leukemia cells, a significant decrease in the ability to compose with colony formation, a significant increase in gamma-yH2AX nuclear foci of MA9 in vitro, reduced tumor load in the MA9-driven leukemia mouse model, and prolonged host survival [100]. After knockdown of MOF, human myeloid leukemia cells U937 and HL-60 proliferated and increased CD93 expression [101]. Chidamide inhibits autophagy in MM cells (H929 and RPMI8226) by downregulating histone deacetylase sirtuin (SIRT) 1 and upregulating acetyltransferase hMOF, contributing to an increase in the overall level of H4K16ac [54]. It is reported that low levels of H4K5Ac were detected in AML patients’ primary cells, which might be due to the low survival of AML patients [102]. With the catalytic subunit hMOF, the NSL complex mediates the acetylation of the H4K16, H4K5, and H4K8 residues, which indicate the important role of the NSL complex in the development and progression of MLL-AF9 (MA9) leukemia, human myeloid leukemia, and AML.

## 5. OGT/TET Complex

The ten-eleven translocation (TET) protein is an α-ketoglutarate- and Fe^2+^-dependent dioxygenase with enzymatic activity catalyzing the hydroxylation of DNA 5-methylcytosine (5-mC) [103], which can drive DNA demethylation and further regulate gene transcription, including the so-called sry-related HMG box (SOX), runt-related transcription factor 1 (RUNX1), phosphatase and tensin homolog (PTEN), and p16, thus playing important roles in biological processes such as embryonic development, hematopoietic stem cell differentiation, malignant cell proliferation, and cell cycle [104]. The TET protein family contains three members, TET1, TET2, and TET3, all with the same catalytic activity of converting 5-mC to 5-hydroxymethylcytosine (5-hmC) but with significant cell/organ specificity in expression, with TET1 being mainly expressed in embryonic stem cells, TET2 being more abundant in the haematopoietic system, and TET3 being widely expressed in the cerebellum, cortex, and hippocampus of the nervous system [105]. Numerous experimental and clinical data confirm that abnormalities in TET protein expression and function are closely associated with the development and progression of hematological malignancies [106]. The TET1 protein was first identified in patients with AML with chromosomal t (10; 11) (q22; q23) translocation, and TET1 is fused to the MLL gene [107]. Related studies have shown that TET1 binding to MLL leads to the upregulation of TET1 expression (overall increase in 5-hmC levels) and subsequent activation of signaling pathways such as Homeobox A (HOXA) 9, Meis homeobox 1 (Meis1), and pre-B-cell leukemia homeobox 3 (PBX3), which in turn promote the development and progression of MLL rearrangement leukemia [108]. Only a few reports have observed TET1 mutations, such as AML, CLL, and T-ALL [109]. Among the TET family, TET2 is the most widely studied member of hematological malignancies and is a common mutated gene in hematological malignancies [110], and it is thought that TET2 mutations/deletions lead to abnormal 5-mC levels and consequently to myeloid and lymphoid hematological malignancies [111]. However, the relationship between TET3 function and hematological malignancies has been unclear until now, with TET3 mutations only occasionally found in CLL [112] and peripheral T-cell lymphoma (PTCL) [113]. Recent studies have shown that enzymes of TETs can form a complex with OGT to perform cellular functions. For example, it has been reported that TET2 and TET3 can interact with OGT to form stable TET2/OGT and TET3/OGT complexes based on the interaction of the TPR domain of OGT with the H domain of the catalytic domain of TET proteins [114]. The interaction between OGT and TET proteins does not appear to regulate the expression of TET and its enzymatic activity [112], but it is able to recruit OGT to chromatin and subsequently cause *O*-GlcNAcylation of histones to regulate gene expression [58]. It is reported that the loss of function of TET2 is observed for 10–20% of patients with MDS/myeloproliferative neoplasm (MPN) [115], 40–50% of patients with CMML, and 10–20% of patients with AML [116], and this confirms that TET2 mutations are associated with poor outcome in intermediate risk AML [117]. In addition, the deletion of TET2 has been observed for patients with malignant lymphoma [118], and decreased levels of 5-hmC and increased levels of 5-mC have been observed for patients with TET2 mutations in lymphoma, AML, chronic myelomonocytic leukemia (CMML), and MDS [119]. This suggests that TET2, as a tumor suppressor, can influence the development of hematological malignancies by regulating DNA methylation levels. However, in hematological malignancies, the loss of function of the TET2 protein does not directly lead to malignant tumor formation. Related studies have shown that TET2 and other epigenetic modifier genes act synergistically and in turn influence the development of hematological malignancies [120]. OGT/TET acts as an epigenetic scaffold that recruits HCF-1 and affects the integrity of the H3K4 methyltransferase SET1/COMPASS complex by inducing HCF-1 *O*-GlcNAcylation, which in turn promotes H3K4me3 [57]. Triptolide has been found to reduce the protein levels of c-Myc and vascular endothelial growth factor A (VEGFA) by inhibiting H3K4me3, thereby inducing growth inhibition in MM cells (KM3) as well as apoptosis in KM3 cells [121]. In contrast, Wong et al. suggested that high levels of H3K4me3 could positively regulate the oncogenic potential of leukemic stem cells and promote the production of MLL-rearranged AML in mice and humans [122]. In addition, the depletion of EZH2 combined with TET2 deficiency significantly accelerates the development of myelodysplastic disorders, including MDS and MDS/MPN overlap disorders [123]. Moreover, abnormal tumor cell metabolism may alter the *O*-GlcNAcylation of TET2 proteins, thereby affecting their stability. Conversely, the absence of the TET function in tumors may affect the cytosolic and/or cytoplasmic distribution of OGT, which in turn may affect the stability of tumor suppressors and oncogenes such as p53 [124] and MYC [125]. This implies that the OGT/TET complex may influence the development of hematological malignancies by regulating epigenetic modifications, including DNA methylation and histone methylation associated with the positive regulation of gene expression.

## 6. PR-DUB Complex

Additional sex combs-like (ASXL) 1, an epigenetic modulator, is a subunit of Polycomb repressive deubiquitinase (PR-DUB) complex, which also include OGT, HCF-1, BRCA1-associated protein 1 (BAP1), ASXL2, ASXL3, lysine demethylase 1B (KDM1B), MBD5/6, MLL3, as well as FOXK1/2 [126]. PR-DUB affects apoptosis, cell proliferation, and myeloid differentiation by removing Polycomb repressive complex 1 (PRC1)-mediated H2AK119 ubiquitin (ub) 1 and altering the chromatin structure to regulate gene transcription such as B-cell lymphoma-2 (Bcl2) and Mcl1 [59,60,61]. OGT improves ASXL1 stability by inducing *O*-GlcNAcylation at the ASXL1 Ser199 site [127], while ASXL1 interacts with and activates BAP1 [66]. It is reported that ASXL1 is mutated in numerous myeloid malignancies, including MDS, AML, and CMML [128,129], and ASXL1 mutations have been shown to be positively associated with poor prognosis in patients with MDS and AML [130,131]. The ASXL1 mutation occurs at the C-terminus, generating a C-terminally truncated ASXL1 mutant [132,133], which retains the DEUBAD binding domain and facilitated the catalytic activity of BAP1 and promotes H2AK119ub1 deubiquitination, which in turn drives myeloid leukemogenesis [134]. Such as, the mutant ASXL1 (ASXL1-MT)/BAP1 complex can impair the multidirectional differentiation of HSPCs (except to monocytes) and promote RUNX1-ETO-induced murine c-Kit+ progenitor cell leukemogenesis by removing H2AK119ub, which drives the upregulation of the HOXA gene and interferon regulatory factor 8 (IRF8) [135]. Moreover, the mutant ASXL1 (ASXL1-MT)/BAP1 complex promotes MLL rearrangement leukemia progression by reducing the upregulation of HOXA5, HOXA7, HOXA9, and IRF8 induced by H2AK119ub1 [136]. Therefore, we speculate that the effect of PR-DUB on the progression of hematological malignancies may be dependent on H2AK119 deubiquitination activity. 

## 7. Small Molecules Targeting OGT

With the discovery of the abnormal high expression of OGT in numerous diseases including hematological malignancies, small molecules targeting OGT to correct the upregulated *O*-GlcNAcylation has attracted increasing attention [137]. 

It is reported that numerous microRNA could target OGT as well as activate or inhibit OGT-mediated *O*-GlcNAcylation, which are further functions in cancer progression. Furthermore, miR-7-5p binds with the 3′ UTR domain of OGT, represses OGT expression, and inhibits lung cancer cell (H522, H460, and H1299 cell lines) proliferation and cancer metabolism [138]. In addition to miR-485-5p, miR-101 targets OGT-mediated *O*-GlcNAcylation, further inhibiting colorectal cancer progression [139,140]. MicroRNA24 is also an OGT regulator, reduces the *O*-GlcNAcylation of FOXA1 and c-Myc, and prevents breast cancer cells and mouse hepatoma cell invasion, respectively [141,142]. Moreover, miR-424-5p could target OGT, further inhibiting the progression of liver cancer and clear cell renal cell carcinoma [143]. In is mentioned that miR-146a-5p [144], microRNA-423-5p [145], miR-7, miR-15a [146], mi-140 [147], and miR-483 [148] are identified to be negative regulators of OGT, and it functions in indicated cancer proliferation through inhibiting OGT.

On the other hand, three main classes of inhibitors have been reported, which are UDP backbone-type inhibitors, dual-substrate inhibitors, and small-molecule inhibitors obtained from HTS [149]. With disadvantages such as low transmembrane capacity, which affect other glycosylation processes and poor water solubility, UDP backbone-type inhibitors and dual-substrate inhibitors are often used as laboratory tools for drug studies. Whereas HTS-based small molecule inhibitors are diverse and have numerous structures, most of them act on the C-terminal catalytic pocket of OGT with a single mode of action [150]. OMSI-1 is a cell-permeable OGT inhibitor [151]. It was found that chemoresistance was associated with increased levels of *O*-GlcNAcylated proteins and that the OGT inhibitor OSMI-1 enhanced the killing effect of adriamycin on HL-60 cells and primary AML cells in patients with relapsed or resistant disease [152]. Based on the critical role of OGT in hematological tumors, we look forward to more experimental and clinical studies targeting OGT in the treatment of hematological malignancies.

## 8. Conclusions and Perspectives

**Figure 2:**
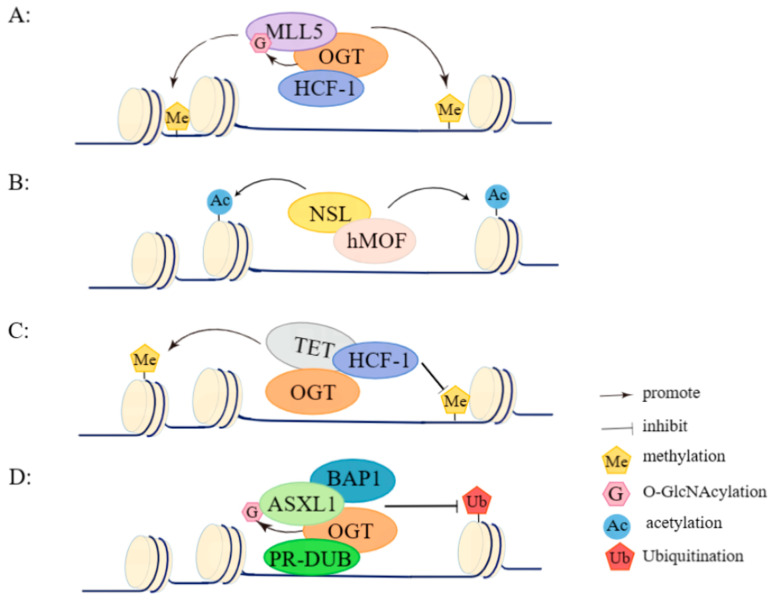
OGT complexes influence the development and progression of hematological tumors by regulating epigenetic modifications. (**A**) The OGT/HCF-1 complex recruits MLL5 and regulates H3K4me by inducing MLL5 *O*-GlcNAcylation. (**B**) The NSL complex promotes histone acetylation using hMOF as the catalytic subunit. (**C**) OGT/TET complex promotes DNA demethylation and histone methylation through the recruitment of HCF-1. (**D**) The core catalytic subunit of the PR-DUB complex, AXSL1, has increased stability catalyzed by OGT, which in turn cooperates with BAP1-mediated histone deubiquitination.

Cancer development and progression are often accompanied by the overexpression of OGT and abnormal overall *O*-GlcNAcylation, which may drive the life course of cancer cell survival, proliferation, metastasis, and invasion. In cells, OGT is able to assemble with a variety of proteins to form complexes that are involved in the regulation of epigenetic modifications (as shown in Figure 2) to mediate the expression of related genes, thereby influencing the development of hematological malignancies. This review focuses on the complexes formed with the involvement of OGT and their role in the development of hematological malignancies.

We provide a more comprehensive summary of OGT complexes in which OGT and binding proteins interact and coordinate with each other. This resource will provide new ideas for cancer therapy. Considering the existence of multiple substrate proteins of OGT, the current research on OGT complexes so far remains relatively limited, but subsequent studies can provide multiple ideas for tumor therapies in greater depth. This potential rests particularly with genes involved in epigenetic inheritance and inhibitors of related complexes.

## Figures and Tables

**Table 2:** OGT is involved in assembled complexes as well as their biological functions.

	Binding Protein	Complex	Biological Function(s)	Reference(s)
OGT	HCF-1	OGT/HCF-1 complex	Regulates the epigenetic modifications and performs related functions by forming temporary complexes in concert with other proteins, such as MYC and MLL5.	[52,53,54,55]
	MOF, NSL1, NSL2, NSL3, MCRS2, WDR5, OGT, HCF-1	NSL complex	Catalyzes histone H4K16/K5/K8 acetylation.	[56]
	TET.	OGT/TET complex	Recruits OGT to chromatin and subsequently causes the *O*-GlcNAcylation of histones to regulate gene expression, and OGT-TET2/3 *O*-GlcNAcylates HCF-1 and regulates SET1/COMPASS-mediated H3K4me3.	[57,58]
	HCF-1, BAP1, ASXL2, ASXL3, KDM1B, MBD5/6, MLL3, FOXK1/2	PR-DUB complex	Catalyzes H2AK119 deubiquitination.	[59,60,61]
	OGA	*O*-GlcNAczyme complex	Regulates signal transduction, glucose metabolism, cell proliferation, and apoptosis, as well as other basic cellular processes.	[62,63,64,65]
	HUWE1	OGT/HUWE1 complex		
	Rev-erbα	OGT/Rev-erbα complex		

## Data Availability

Data sharing is not applicable to this article.
